# Author Correction: High p16 expression and heterozygous *RB1* loss are biomarkers for CDK4/6 inhibitor resistance in ER^+^ breast cancer

**DOI:** 10.1038/s41467-022-34580-3

**Published:** 2022-11-14

**Authors:** Marta Palafox, Laia Monserrat, Meritxell Bellet, Guillermo Villacampa, Abel Gonzalez-Perez, Mafalda Oliveira, Fara Brasó-Maristany, Nusaibah Ibrahimi, Srinivasaraghavan Kannan, Leonardo Mina, Maria Teresa Herrera-Abreu, Andreu Òdena, Mònica Sánchez-Guixé, Marta Capelán, Analía Azaro, Alejandra Bruna, Olga Rodríguez, Marta Guzmán, Judit Grueso, Cristina Viaplana, Javier Hernández, Faye Su, Kui Lin, Robert B. Clarke, Carlos Caldas, Joaquín Arribas, Stefan Michiels, Alicia García-Sanz, Nicholas C. Turner, Aleix Prat, Paolo Nuciforo, Rodrigo Dienstmann, Chandra S. Verma, Nuria Lopez-Bigas, Maurizio Scaltriti, Monica Arnedos, Cristina Saura, Violeta Serra

**Affiliations:** 1 Experimental Therapeutics Group, Vall d’Hebron Institute of Oncology, Barcelona, Spain; 2 Breast Cancer and Melanoma Group, Vall d’Hebron Institute of Oncology, Barcelona, Spain; 3 Department of Medical Oncology, Hospital Vall d’Hebron, Barcelona, Spain; 4 Oncology Data Science Group, Vall d’Hebron Institute of Oncology, Barcelona, Spain; 5 Institute for Research in Biomedicine (IRB Barcelona), Barcelona, Spain; 6 Research Program on Biomedical Informatics, Universitat Pompeu Fabra, Barcelona, Spain; 7 Translational Genomics and Targeted Therapies in Solid Tumors, August Pi i Sunyer Biomedical Research Institute (IDIBAPS), Barcelona, Spain; 8 Service de Biostatistique et d’Epidémiologie, Gustave Roussy, Villejuif, France; 9 Oncostat U1018, Inserm, University Paris-Saclay, Villejuif, France; 10 Bioinformatics Institute (A*STAR), Singapore, Singapore; 11 Medica Scientia Innovation Research (MedSIR), Barcelona, Spain; 12 The Breast Cancer Now Research Centre, London, UK; 13 Preclinical Modelling of Pediatric Cancer Evolution Group, The Institute of Cancer Research, London, UK; 14 Translational Molecular Pathology, Vall d’Hebron Institute of Research (VHIR), Barcelona, Spain; 15 Novartis Pharmaceuticals, East Hanover, NJ USA; 16 Genentech, Inc., South San Francisco, California, USA; 17 Breast Biology Group, Manchester Breast Centre, Manchester, UK; 18 Cancer Research UK, Cambridge, UK; 19 CIBERONC, Vall d’Hebron Institute of Oncology, Barcelona, Spain; 20 Growth Factors Laboratory, Vall d’Hebron Institute of Oncology, Barcelona, Spain; 21 Department of Biochemistry and Molecular Biology, Universitat Autònoma de Barcelona, Barcelona, Spain; 22 IMIM (Hospital del Mar Medical Research Institute), Barcelona, Spain; 23 Institució Catalana de Recerca i Estudis Avançats (ICREA), Barcelona, Spain; 24 University of Barcelona, Barcelona, Spain; 25 Department of Medical Oncology, Hospital Clinic, Barcelona, Spain; 26 SOLTI Breast Cancer Research Group, Barcelona, Spain; 27 Department of Oncology, IOB Institute of Oncology, Barcelona, Spain; 28 Molecular Oncology Group, Vall d’Hebron Institute of Oncology, Barcelona, Spain; 29 School of Biological Sciences, Nanyang Technological University, Singapore, Singapore; 30 Department of Biological Sciences, National University of Singapore, Singapore, Singapore; 31 Departments of Pathology and Human Oncology and Pathogenesis Program, Memorial Sloan-Kettering Cancer Center, New York, USA; 32 Department of Medical Oncology, Gustave Roussy, Villejuif, France; 33 Inserm Unit U981, Villejuif, France

**Keywords:** Breast cancer, Predictive markers, Breast cancer, Cancer models

Correction to: *Nature Communications* 10.1038/s41467-022-32828-6, published online 07 September 2022

The original version of the Supplementary Information associated with this Article included an incorrect Supplementary Information file, in which tracked changes from revision were present. The HTML has been updated to include a corrected version of the Supplementary Information; the original incorrect version can be found as Supplementary Information associated with this Correction.

In the original version of the published article, the Acknowledgements section omitted a grant number. The correct Acknowledgements paragraph includes the following: This study has been supported by the Susan G. Komen Foundation (CCR15330331) and by the Catalan Agency AGAUR (2017 SGR 540).

This has now been corrected both in the HTML and the PDF versions of the article.

## Supplementary information

Supplementary Information

Original Supplementary Information

