# Supplementary material for: High p16 expression and heterozygous RB1 loss are biomarkers for CDK4/6 inhibitor resistance in ER+ breast cancer
Source: Nat Commun. 2022 Sep 7;13:5258. doi: 10.1038/s41467-022-32828-6 (PMC9452562; doi:10.1038/s41467-022-32828-6)
Supplement: Supplementary file 1 — Supplementary Info [file 41467_2022_32828_MOESM1_ESM.pdf]

SUPPLEMENTARY FIGURE AND TABLES

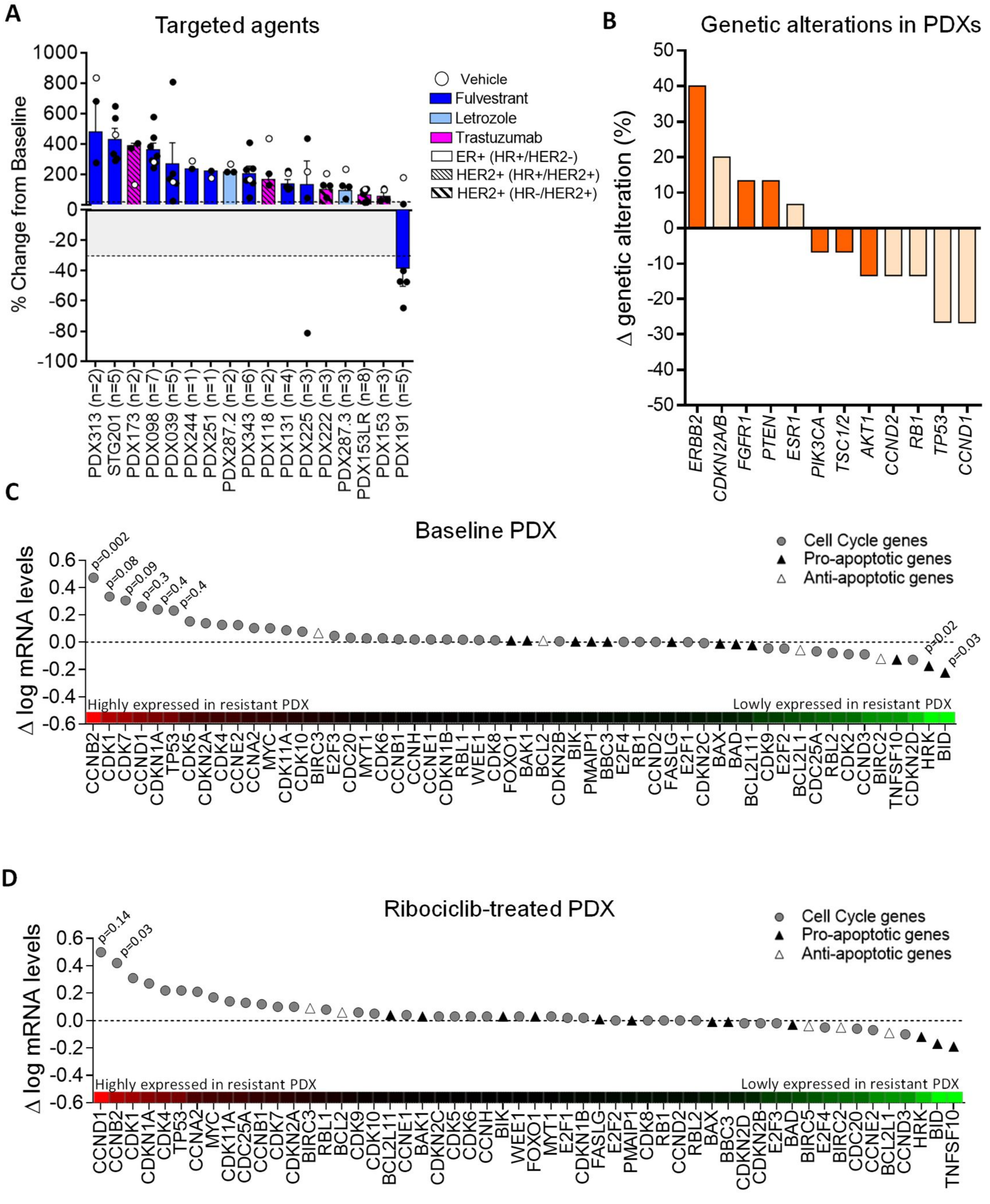

Fig. S1. Antitumor efficacy of others targeted therapies and genomic and transcriptomic analysis of PDXs. (A) Waterfall plot representing the growth of 12 ER$^+$ and 5 HER2$^+$ PDXs treated with endocrine therapy (10 mg/mouse fulvestrant or 20 mg/kg letrozole) or 10mg/kg trastuzumab (bars) and vehicle (white circles). The number of tumors (black circles) treated per model is indicated in the brackets (n). The percentage change from the initial volume is shown at day 15 of treatment. Data represent mean values ± SEM. Dashed lines indicate the range of PD (>20%), SD (20% to -30%) and PR/CR (<-30%). (B) Incidence of alterations in 12 genes related to PI3K

and cell cycle, analyzed by IMPACT™ in untreated ribociclib-sensitive *vs*. ribociclib-resistant PDXs. For this analysis copy number amplifications (lr≥2) and deleterious mutations (missense putative drivers, frameshift, and splice mutations) were considered. Different colors indicate the specific gene-related pathways. (C) and (D) mRNA levels of 54 cell cycle and apoptosis genes in ribociclib-resistant (n=12 tumors) *vs*. ribociclib-sensitive (n=5 tumors) PDXs measured by RT-qPCR in untreated (baseline) or treated with 75 mg/kg ribociclib for 12 days. Gene expression was normalized to housekeeping genes (*ACTB* and *GAPDH*) and, mean-centered data is provided. Symbols indicate the specific gene-related pathways. Mean values and unpaired parametric *t*-test two-tailed *p*-value are indicated.

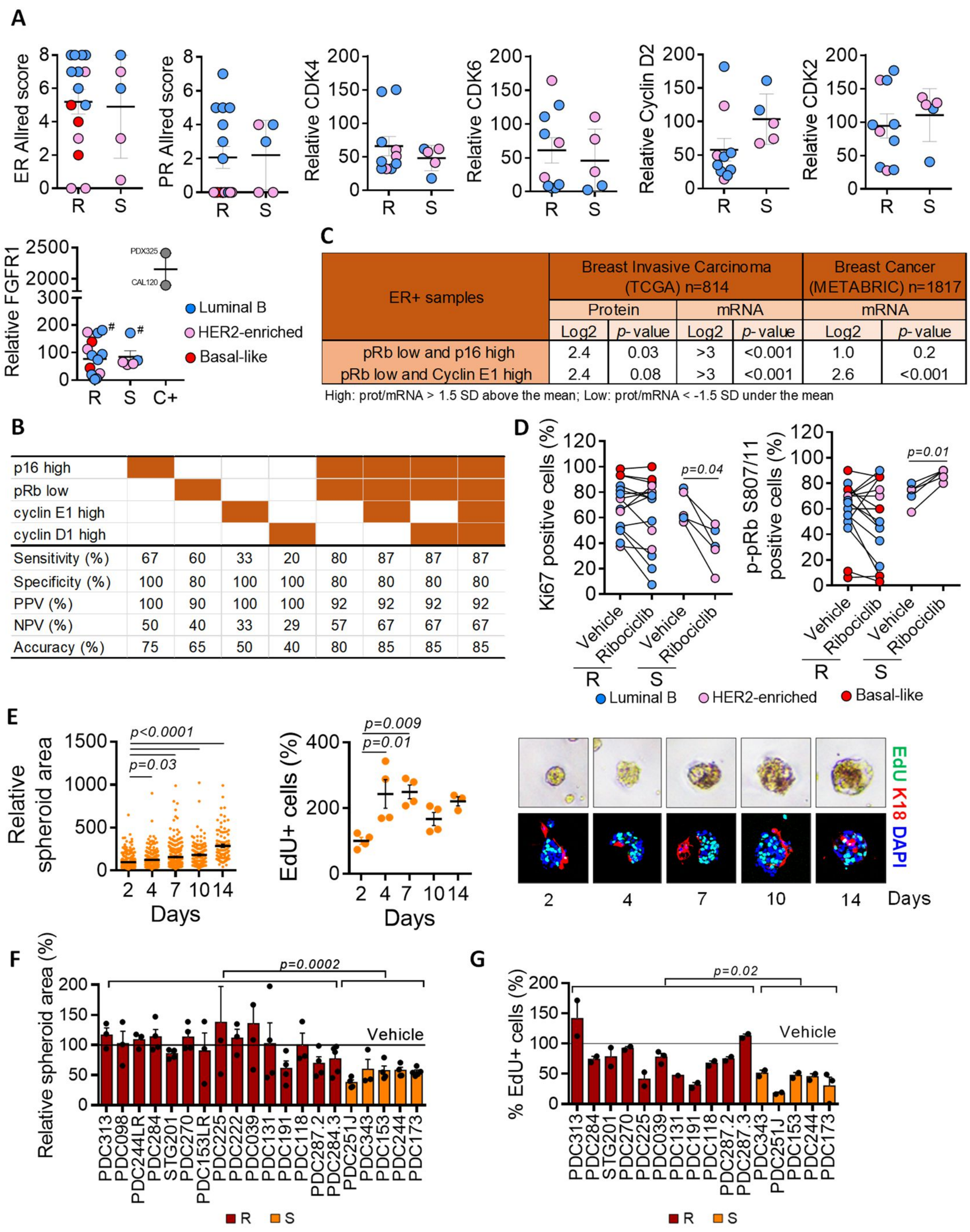

ER+ samples	Breast Invasive Carcinoma (TCGA) n=814				Breast Cancer (METABRIC) n=1817	
	Protein		mRNA		mRNA	
	Log2	*p*-value	Log2	*p*-value	Log2	*p*-value
pRb low and p16 high	2.4	0.03	>3	<0.001	1.0	0.2
pRb low and Cyclin E1 high	2.4	0.08	>3	<0.001	2.6	<0.001

High: prot/mRNA > 1.5 SD above the mean; Low: prot/mRNA < -1.5 SD under the mean

p16 high	■				■	■	■	■
pRb low		■			■	■	■	■
cyclin E1 high			■			■		■
cyclin D1 high				■			■	■
Sensitivity (%)	67	60	33	20	80	87	87	87
Specificity (%)	100	80	100	100	80	80	80	80
PPV (%)	100	90	100	100	92	92	92	92
NPV (%)	50	40	33	29	57	67	67	67
Accuracy (%)	75	65	50	40	80	85	85	85

Fig. S2. Expression levels of several proteins in PDXs and spheroid area and EdU-incorporation analysis in PDCs *ex-vivo* cultures. (A) Quantification of the indicated proteins in untreated PDX (n=23) detected by IHC (ER and PR) or Western blot (CDK4, CDK6, cyclin D2, CDK2 and FGFR1) over two independent experiments. For FGFR1, expression levels were compared to two positive controls (C+; PDX325 and CAL120 cell line), which harbored *FGFR1* amplification. Different colors indicate the PDXs intrinsic subtype and hashtags indicate models harboring gene amplification. Data are represented as mean values ± SEM. R, resistant; S, sensitive. (B) Prediction analysis of the indicated biomarker(s) to classify a PDX as resistant or sensitive to

ribociclib based on their expression levels. High p16 mean value expression score ≥ 2+, low pRb mean value expression score ≤ 2+ and high cyclin E1/D1 mean value Allred score > 4/6. (C) Co-occurrence of altered pRb and p16 or pRb and cyclin E1 expression levels in two cohorts of ER^+ breast. Data and statistical analysis were extracted from the cBioportal (www.cBioportal.org). The cut-off for high versus low protein/mRNA levels is indicated. OR: odd´s ratio; prot: protein; SD: standard deviation. (D) Analysis of Ki67 (left graph) and phospho-pRb S807/811 (right graph) in vehicle and 14 days ribociclib-treated PDXs. For illustration purposes, only the mean value of each PDX was plotted; however, for the statistical analysis all technical replicates were used. Two-tailed *p*-values are based on Mann-Whitney U test are indicated. Different colors represent the PDXs intrinsic subtype. R, resistant; S, sensitive. (E) Relative spheroid area (left graph) or percentage of Edu-positive cells (right graph) in untreated PDC287.3 for the indicated time. Data are represented as mean values of three independent experiments ± SEM. Two-tailed *p*-values are based on one-way ANOVA test with Tukey´s method correction are shown. Underneath pictures show representative bright field or confocal microscopy images of PDC287.3 at different time points. Magnification 40x. Quantification of the relative spheroid area (F) or percentage of EdU-positive cells (G) in the indicated PDCs after treatment with 1 μM ribociclib in *ex vivo* cultures for 7 days. Data are represented as mean values of three independent experiments ± SEM. Two-tailed *p*-values are based on the one-way ANOVA test with Tukey´s method correction are shown. Black lines indicate the vehicle conditions. R; resistant; S; sensitive, according to the *in vivo* ribociclib anti-tumor activity.

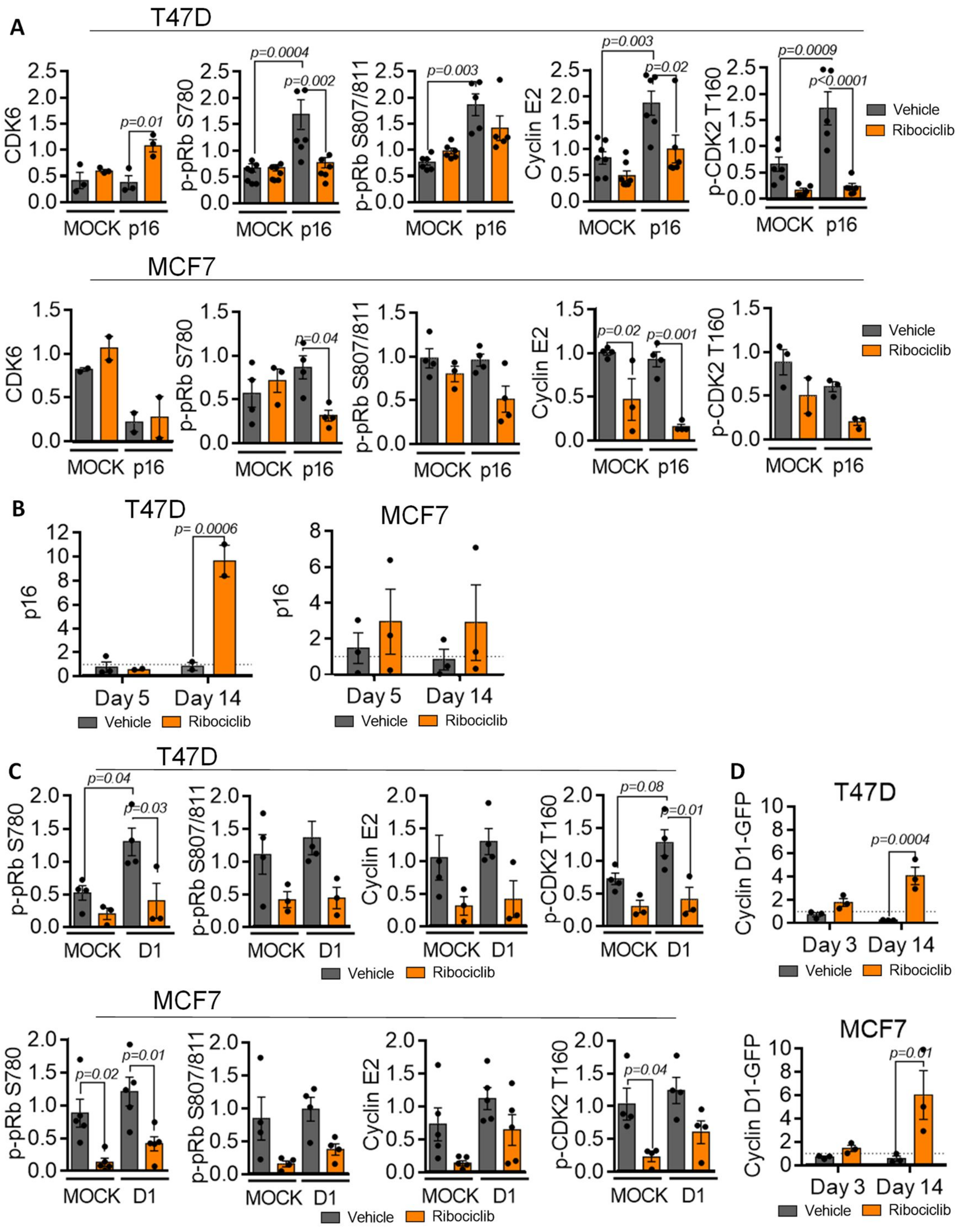

Fig. S3. Validation of p16, pRb and cyclin D1 as biomarkers of resistance to ribociclib in ER⁺ cell lines and patients. (A) Quantification of the expression levels of indicated proteins relative to tubulin analyzed by Western blot in T47D- and MCF7-p16 untreated or treated with 0.5 μM of ribociclib for 24 hours (n=3 independent experiments). Data are represented as mean values ± SEM. Unpaired parametric *t*-test two-tailed *p*-values are indicated. (B) Quantification of the expression levels of the indicated proteins relative to tubulin in three independent enrichment experiments using T47D- or MCF7-p16 cells seeded at 1:20 dilution with MOCK-transfected cells

and treated with 0.5 μM ribociclib or vehicle for the indicated time (n=2 independent experiments). Data are represented as mean values ± SEM. Unpaired parametric *t*-test two-tailed *p*-values are indicated. (C) Quantification of the expression levels of the indicated proteins relative to tubulin analyzed by Western blot in T47D-cyclin D1 and MCF7-cyclinD1 untreated or treated with 0.5 μM of ribociclib for 24 hours (n=3 independent experiments). Data are represented as mean ± SEM. Unpaired parametric *t*-test two-tailed *p*-values are indicated. (D) Quantification of the expression levels of the indicated proteins relative to tubulin in an enrichment experiment using T47D- or MCF7-cyclin D1 cells seeded at 1:20 dilution with MOCK-transfected cells and treated with 1 μM ribociclib or vehicle for the indicated time (n=3 independent experiments). Data are represented as means ± SEM. Unpaired parametric *t*-test two-tailed *p*-values are indicated.

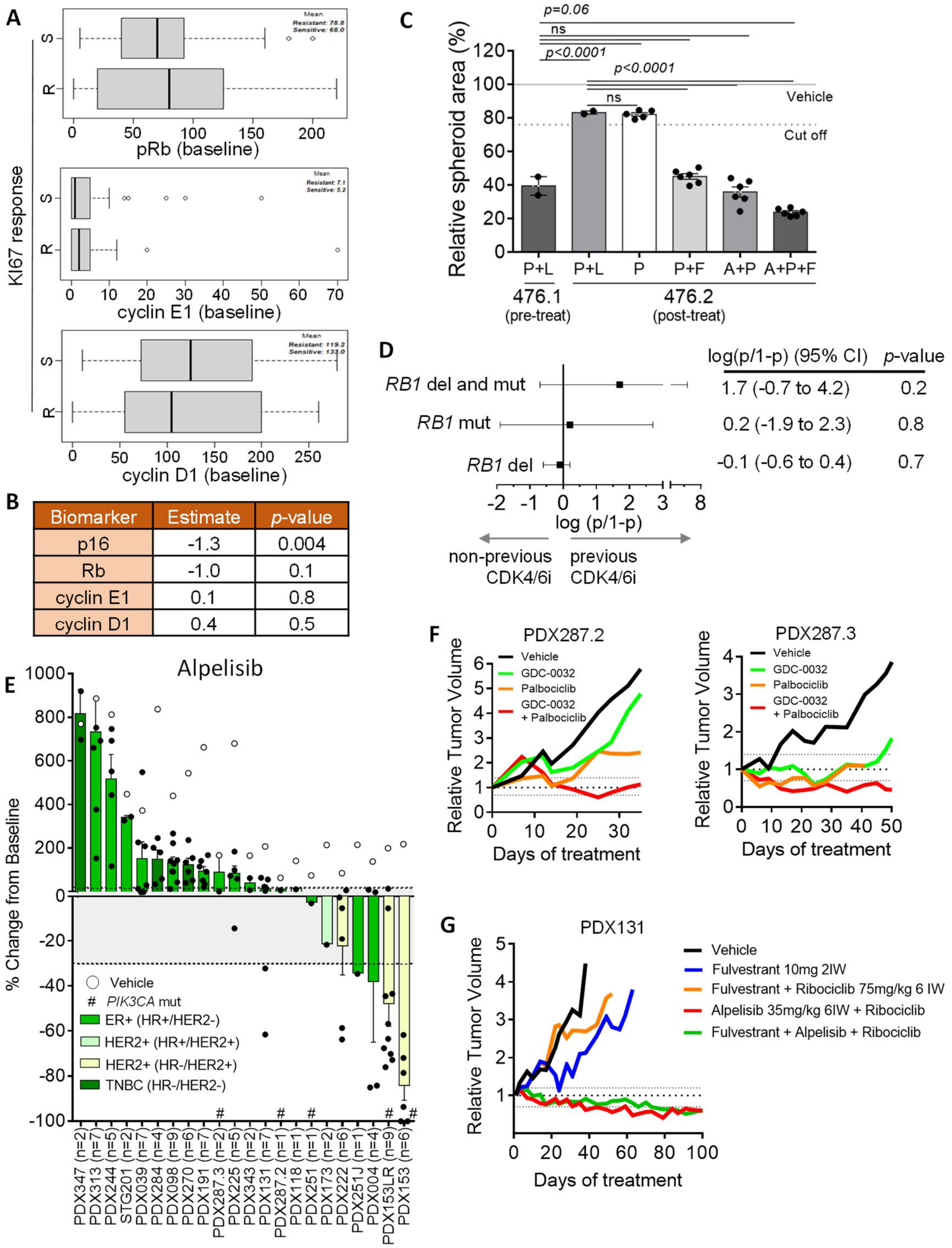

Biomarker	Estimate	*p*-value
p16	-1.3	0.004
Rb	-1.0	0.1
cyclin E1	0.1	0.8
cyclin D1	0.4	0.5

Fig. S4. Validation of RB1 homozygous loss as predictor of acquired resistance to ribociclib and antitumor efficacy of PI3K inhibitor in PDXs. (A) Box and whisker plot showing a logistic model to evaluate the effect of pRb (upper), cyclin E1 (middle) and cyclin D1 (bottom) on the response to abemaciclib in in the ABC-POP trial tumor samples. Box represents the median and the 25th and 75th percentiles, whiskers show the largest and smallest value. The mean value of each subgroup is indicated. (B) Multivariant logistic regression of complex biomarkers. Two-tailed *p*-values are

shown. (C) Relative spheroid area in PDX476.1 treated with 500 nm palbociclib and PDC476.2 after treatment with 500 nM palbociclib as single-agent or combined with 100 nM fulvestrant and/or 2.5 µM alpelisib in *ex vivo* cultures for 7 days. Data are presented as means of three independent experiments ± SEM. Two-tailed *p*-values are based on the one-way ANOVA test with Tukey´s method correction compared with the vehicle (black line) are indicated. The dashed line indicates the optimal cut-off established in Figure 3E. (D) Association between *RB1* alterations (only mutation, only deletion or both) and prior exposure to CDK4/6 inhibitors across metastatic breast cancer patients. The black squares represent the logit values. Multivariable logistic regression two-tailed *p*-value and the 95% confidence intervals (CI; horizontal segment represents) for each test are shown. (E) Waterfall plot representing the growth of 23 PDX treated with alpelisib 35 mg/kg (bars) or vehicle (white circles). The number of tumors (black circles) treated per model is indicated in the brackets (n). The percentage change from the initial volume is shown at day 35 of treatment. Dashed lines indicate the range of PD (>20%), SD (20% to -30%) and PR/CR (<-30%). Hashtags indicate models harboring mutations in *PIK3CA.* Data is represented as mean values ± SEM. Relative tumor growth of PDX287.2 and PDX287.3 (F) or PDX131 (G) treated with the indicated drugs and time. Dashed lines indicate the range of PD (>1.2), SD (1.2 to -0.7) and PR/CR (<-0.7).

		Molecular Subtype		ER status		PR status		HER2 status		KI67 (%)	
Sample ID	Origin	Patient	PDX	Patient	PDX	Patient	PDX	Patient	PDX	Patient	PDX
4	Metastatic (skin)	ER$^+$/HER2$^-$	ER$^+$/HER2$^-$	pos	pos	neg	pos	neg	nd	50	25
39	Metastatic (skin)	ER$^+$/HER2$^-$	ER$^+$/HER2$^-$	pos	pos	neg	neg	neg	nd	50	80
98	Primary	ER$^+$/HER2$^-$	ER$^+$/HER2$^-$	pos	pos (low)	neg	neg	neg	neg	70	75
118	Metastatic (skin)	nd	ER$^+$/HER2$^+$	nd	pos	nd	neg	nd	pos	nd	50
131	Metastatic (skin)	nd	ER$^+$/HER2$^-$	nd	pos	nd	pos	nd	nd	nd	50
153	Primary	HER2$^+$	HER2$^+$	neg	neg	neg	neg	pos	pos	40	35
153LR	Primary	HER2$^+$	HER2$^+$	neg	neg	neg	neg	pos	nd	40	75
173	Primary	ER$^+$/HER2$^+$	ER$^+$/HER2$^+$	pos	pos	pos	pos	pos	pos	20	60
191	Primary	ER$^+$/HER2$^-$	ER$^+$/HER2$^-$	pos	pos	pos	pos	neg	nd	30	75
STG201	Metastatic (nd)	nd	ER$^+$/HER2$^-$	nd	pos (low)	nd	neg	nd	nd	nd	95
222	Primary	HER2$^+$	HER2$^+$	neg	neg	neg	neg	pos	pos	80	85
225	Metastatic (skin)	nd	ER$^+$/HER2$^-$	nd	pos (low)	nd	neg	nd	nd	nd	50
244	Metastatic (skin)	nd	ER$^+$/HER2$^-$	nd	pos	nd	pos	nd	nd	nd	75
244LR	Metastatic (skin)	nd	ER$^+$/HER2$^-$	nd	pos	nd	pos	nd	nd	nd	75
251J	Primary	ER$^+$/HER2$^+$	ER$^+$/HER2$^+$	pos	pos	neg	neg	pos	pos	50	45
251	Primary	ER$^+$/HER2$^-$	ER$^+$/HER2$^-$	pos	pos	pos	pos	neg	neg	30	50
270	Primary	ER$^-$/HER2$^-$	ER$^-$/HER2$^-$	neg	neg	neg	neg	neg	nd	70	85
284	Primary	ER$^-$/HER2$^-$	ER$^-$/HER2$^-$	neg	neg	pos	neg	neg	neg	45	75
287.2	Primary	ER$^+$/HER2$^-$	ER$^+$/HER2$^-$	pos	pos	pos	pos	neg	neg	80	40
313	Metastatic (skin)	nd	ER$^+$/HER2$^-$	nd	pos	nd	neg	nd	neg	nd	95
287.3	Primary	ER$^+$/HER2$^-$	ER$^+$/HER2$^-$	pos	pos	pos	pos	neg	neg	80	65
343	Metastatic (breast)	ER$^+$/HER2$^-$	ER$^+$/HER2$^-$	pos	pos	pos	pos	neg	nd	60	70
347	Primary	ER$^-$/HER2$^-$	ER$^-$/HER2$^-$	neg	neg	neg	neg	neg	neg	80	80

Table S1. Status ER, PR, HER2 and percentage of KI67-positive cells in matched patients and PDXs tumors**.** Abbreviations: pos (positive); nd (not determined).

Sample ID	Biopsy origin	Patient TNM	Treatments before biopsy
4	Metastasis	T2N0M1	NeoAdj / Adj / Met: NA
39	Metastasis	M1	NeoAdj /Adj: NA; Met: L, T, Ex, F + R, Be, Ct (5 lines)
98	Primary	T1N1M0	NeoAdj: Ct (2 lines); Adj: NA
118	Metastasis	NA	NeoAdj: NA; Adj: Ct, T; Met: H + Ct (6 lines), R, La + H, HS
131	Metastasis	M0	NeoAdj: NA; Adj: Ct (2 lines), T; Met: Ct (3 lines), L, Ex, Ct+Be, Ct+F
153/153LR	Primary	T4N1M0	NeoAdj: Ct+H; Adj: R+H+T+LH, Ct+H+R+LH+L
173	Primary	M1	NeoAdj: NA; Adj: Ct+H+T; Met: NA
191	Primary	NA	NeoAdj / Adj: NA
STG201	Metastasis	NA	NeoAdj / Adj / Met: NA
222	Primary	T2N2M1	NeoAdj / Adj: NA; Met: Ct+H, R+H, Ct+T
225	Metastasis	M1	NeoAdj / Adj: NA; Met: A
244/244LR	Metastasis	M1	NeoAdj / Adj: NA Met: Ct (3 lines), Ct+Be, Ct+R, F+Ev+Ex
251J	Primary	T2N1M0	NeoAdj / Adj: NA
251	Primary	T2N0M0	NeoAdj: NA; Adj: L
270	Primary	T2N0M0	NeoAdj / Adj: NA
284	Primary	T2N0M0	NeoAdj: Ct; Adj: NA
287.2	Primary	T2N1M1	NeoAdj: NA; Adj: Ct, LH+T
313	Metastasis	NA	NeoAdj: Ct (2 lines); Adj: NA; Met: Ct+R, Ct
287.3	Primary	T2N1M1	NeoAdj: NA; Adj: Ct, LH+T
343	Metastasis	M0	NeoAdj: Ct; Adj: R+L-A; Met: P+Ex, Ct. Ct+GD
347	Primary	T3N2M0	NeoAdj: Ct; Adj: NA

Table S2. Clinical information of patients' tumors. Abbreviations: L (letrozole); T (tamoxifen); A (anaztrazole); Ex (exemestano); F (fulvestrant); H (herceptin); La (lapatinib); Ev (everolimus); P (palbociclib); GD (GDC-0032); HS (HSP990); LH (analogs LHRH); R (radiotherapy); Be (bevacizumab); Ct (chemotherapy varius); nd (not determined); ne (not exist).

	Gene ID	Mut		CVN	
		aa change	allelic fc.	alteration	log ratio
PDX313	*TP53*	p.X187_splice	1.0		
	CCND2			AMP	2.1
	AKT1	p. E17K	1.0		
PDX347	*TP53*	p.E286*	1.0		
	CDK6	p. V45L	0,4		
	TSC2	p. R1706H	0.5		
PDX098	*TP53*	p.R249S	1.0		
	RB1	p. F721fs	0.9		
PDX244LR	*TP53*	p.C176R	1.0		
	ESR1	p. Y537S	0.3		
	PTEN			del	-3.0
	CDKN2A			del	-3.3
	CDKN2B			del	-3.3
	RB1	p. L694fs	0.6		
PDX284	*TP53*	p.R110P	1.0		
	CDKN2A			del	-6.1
	CDKN2B			del	-6.1
	CCND1	p. D289N	1.0		
	CCND2			AMP	2.1
STG201	*TP53*	p.M237I	1.0		
	CDKN2A			del	-4.6
	CDKN2B			del	-4.6
	AKT1			AMP	1.5
PDX270	*TP53*	p.S241A	1.0		
	AR	p.G454_G455insG	0.6		
PDX153LR	*TP53*			del	-2.1
	PIK3CA	p. K111E	0.6		
PDX225	*TP53*	p.Q167*	1.0		
	AKT1	p. E17K	0.8	AMP	1.5
	AR			AMP	1.9
PDX222	*TP53*	p.R280G	1.0		
	TSC2	p.S526T	1.0		
	AR			AMP	1.4
PDX039	*TP53*	p.V157I, 0.5	0.5		
	TSC2			del	-7
PDX251	*TP53*	p.Y236C	1.0	del	-2.1
	PIK3CA	p. E545K	0.6		
PDX131	*TP53*	p. Q331fs	1.0		
	ESR1	p. Y537S	0.5		
	CDK6			AMP	1.1
	CCND1			AMP	3.4
	CCNE1			AMP	1.1
	AR	p.57_60del	0.2		
PDX191	*FGFR1*			AMP	2.7
	CCND1			AMP	3.0
PDX118	nd				
PDX287.2	*TP53*	p. T256fs	1.0		
	PIK3CA	p.H1047R	0.5		
	CCND1			AMP	3.0
PDX004	nd				
PDX287.3	*TP53*	p. T256fs	1.0		
	PIK3CA	p.H1047R	0.5		
	RB1	p. K810N	0.25		
	CCND1			AMP	3.3
	AR	p.457_457del	0.5		
PDX251J	*TP53*	p.E287*	1.0		
	CDKN2A	p.S12*	1.0		
PDX343	*ESR1*			AMP	1.1
	FGFR1	p. W37C	0.95	AMP	3.2
	PIK3CA			AMP	1.7
PDX153	*TP53*			del	-2.1
	FGFR1			AMP	1.0
	PIK3CA	p. K111E	0,6		
PDX244	*TP53*	p.C176R	1.0		
	ESR1	p. Y537S	0.3		
	PTEN			del	-3.0
	CDKN2A			del	-4.2
	CDKN2B			del	-4.2
PDX173	*FGFR1*			AMP	1.1

Table S3. PDXs mutations and CVN**.** Abbreviations: AMP (amplification); del (deletion); nd (not detected). Gene names are annotated in italic format.

		Biomarker analysis							Ex vivo analysis	
PDX ID	Clinical subtype	PAM50	IMPACT data	pRb	p16	Cyclin D1	Cyclin E1	Predicted response	Δspheroid area	Predicted response
292	ER+ HER2-	HER2-enriched	11q.13 amp (lr 2.3)	2+	3+	7	5	Resistant	25	Resistant
301	ER+ HER2-	Luminal B		2+	3+	4	3	Resistant	-3	Resistant
346	ER+ HER2-	Luminal B	11q.13 amp (lr 5.1)	3+	2+	6	4	Resistant	-8	Resistant
350	ER+ HER2-	Luminal B		3+	2+	8	0	Resistant	-25	Sensitive
376	ER+ HER2-	Luminal B		3+	2+	7	5	Resistant	22	Resistant
399	ER+ HER2-	Luminal B		2+	1+	7	0	Resistant	-33	Sensitive
406	ER+ HER2-	HER2-enriched		3+	1+	5	3	Sensitive	-41	Sensitive
433	ER+ HER2-	Luminal B	11q.13 amp (lr 2.6)	4+	1+	3	4	Sensitive	-34	Sensitive
446B	ER+ HER2-	Luminal B	*CDKN2A* deepDel (lr -3.0)	4+	0	5	3	Sensitive	-30	Sensitive
450	ER+ HER2-	Luminal B		1+	1+	6	0	Resistant	-1	Resistant
BB3RC31	ER+ HER2-	Luminal B	*FAT1* p.2871fs (allel fc. 0.2)	3+	1+	7	0	Resistant	-3	Resistant
BB6RC39	ER+ HER2+	HER2-enriched		4+	1+	6	0	Sensitive	-25	Sensitive
BB6RC87	ER+ HER2+	HER2-enriched		1+	0	8	4	Resistant	12	Resistant
BB6RC160	ER+ HER2+	HER2-enriched	11q.13 amp (lr 2.9)	4+	0	6	0	Sensitive	-37	Sensitive

Table S4. Complex biomarker validation in 14 additional ER^+ BC PDXs. pRb and p16 score: very strong (4+), strong (3+), moderate (2+) weak (1+) or negative staining (0). Cyclin D1 and cyclin E1 Allred score takes into account the percentage of positive cells (0 to 5) plus the staining intensity (0 to 3). Gene names are annotated in italic format.

GENE ID	ASSAY ID	GENE ID	ASSAY ID	GENE ID	ASSAY ID	GENE ID	ASSAY ID
ACTB	143636	CCNB2	101376	CDK6	tbd	FOXO1	137191
BAD	142965	CCND1	100844	CDK7	101429	GAPDH	141139
BAK1	100068	CCND2	101384	CDK8	101433	HRK	145616
BAX	142318	CCND3	102813	CDK9	tbd	MYC	100977
BBC3	144371	CCNE1	139821	CDKN1A	142319	MYT1	147592
BCL2	100083	CCNE2	144468	CDKN1B	100855	PMAIP1	100739
BCL2L1	100088	CCNH	101394	CDKN2A	148945	RB1	149106
BCL2L11	100096	CDC20	102870	CDKN2B	tbd	RBL1	101543
BID	100122	CDC25A	102820	CDKN2C	111127	RBL2	101547
BIK	145589	CDK1	101406	CDKN2D	110945	TNFSF10	101266
BIRC2	100131	CDK10	tbd	E2F1	102827	TP53	101277
BIRC3	tbd	CDK11A	tbd	E2F2	102830	WEE1	102849
BIRC5	101365	CDK2	101416	E2F3	102834		
CCNA2	102811	CDK4	101418	E2F4	102860		
CCNB1	101373	CDK5	105690	FASLG	145654		

Table S5. qPCR assay IDs. *tbd: not catalog assay available.
