# Supplementary material for: High p16 expression and heterozygous RB1 loss are biomarkers for CDK4/6 inhibitor resistance in ER+ breast cancer
Source: Nat Commun. 2022 Sep 7;13:5258. doi: 10.1038/s41467-022-32828-6 (PMC9452562; doi:10.1038/s41467-022-32828-6)
Supplement: Supplementary file 2 — Reporting Summary [file 41467_2022_32828_MOESM2_ESM.pdf]

nature portfolio

Corresponding author(s): *Double-blind peer review submissions: write DBPR and your manuscript number here instead of author names.*

Last updated by author(s): *YYYY-MM-DD*

Reporting Summary

Nature Portfolio wishes to improve the reproducibility of the work that we publish. This form provides structure for consistency and transparency in reporting. For further information on Nature Portfolio policies, see our Editorial Policies and the Editorial Policy Checklist.

Statistics

For all statistical analyses, confirm that the following items are present in the figure legend, table legend, main text, or Methods section.

n/a	Confirmed	
☐	☒	The exact sample size (*n*) for each experimental group/condition, given as a discrete number and unit of measurement
☐	☒	A statement on whether measurements were taken from distinct samples or whether the same sample was measured repeatedly
☐	☒	The statistical test(s) used AND whether they are one- or two-sided<br>*Only common tests should be described solely by name; describe more complex techniques in the Methods section.*
☒	☐	A description of all covariates tested
☐	☒	A description of any assumptions or corrections, such as tests of normality and adjustment for multiple comparisons
☐	☒	A full description of the statistical parameters including central tendency (e.g. means) or other basic estimates (e.g. regression coefficient) AND variation (e.g. standard deviation) or associated estimates of uncertainty (e.g. confidence intervals)
☐	☒	For null hypothesis testing, the test statistic (e.g. *F*, *t*, *r*) with confidence intervals, effect sizes, degrees of freedom and *P* value noted<br>*Give P values as exact values whenever suitable.*
☒	☐	For Bayesian analysis, information on the choice of priors and Markov chain Monte Carlo settings
☒	☐	For hierarchical and complex designs, identification of the appropriate level for tests and full reporting of outcomes
☐	☒	Estimates of effect sizes (e.g. Cohen's *d*, Pearson's *r*), indicating how they were calculated

Our web collection on statistics for biologists contains articles on many of the points above.

Software and code

Policy information about availability of computer code

Data collection	NIS-Elements 5.10 , Pymol 2.3.2, VMD 1.9.1, and Amber 18 software packages were used for data collection. No custom script was developed.
Data analysis	GraphPad Prism 6.00, R version 3.4.3 and 4.0.3, and ImageJ 1.51 software packages were used.

For manuscripts utilizing custom algorithms or software that are central to the research but not yet described in published literature, software must be made available to editors and reviewers. We strongly encourage code deposition in a community repository (e.g. GitHub). See the Nature Portfolio guidelines for submitting code & software for further information.

Data

Policy information about availability of data

All manuscripts must include a data availability statement. This statement should provide the following information, where applicable:

- Accession codes, unique identifiers, or web links for publicly available datasets
- A description of any restrictions on data availability
- For clinical datasets or third party data, please ensure that the statement adheres to our policy

The annotated DNA sequencing data of the PDX models generated in this study is provided in the Source Data file. Access to the raw sequencing data can be made available for academic research only from the corresponding author upon reasonable request and after completing a Data Transfer Agreement with Memorial Sloan-Kettering Cancer Center (New York, USA). Genomic and clinical data of metastatic breast cancer patients from the Hartwig Medical Foundation (HMF) cohort and ABC-POP trial analyzed used in this manuscript were previously published and requested to the corresponding main authors (doi: 10.1038/s41586-019-1689; 10.1016/j.annonc.2020.08.283). Clinical data generated in this study from metastatic breast cancer patients treated at Vall d´Hebron Hospital are provided in the Source Data file. Clinical data shown in figures 6C and supplementary 2C of this manuscript were obtained from the cBioportal (https://www.cbioportal.org/). The 3D protein and compound structures employed in this manuscript are deposited in the PDB (Protein Data Bank) archive: 2A5E (http://dx.doi.org/10.2210/pdb2a5e/

pdb), 1BIN (http://dx.doi.org/10.2210/pdb1bi7/pdb) and P18IN003 (https://pubchem.ncbi.nlm.nih.gov/compound/9994705). No custom script was generated in this study. Source data are provided with this paper.

Field-specific reporting

Please select the one below that is the best fit for your research. If you are not sure, read the appropriate sections before making your selection.

☒ Life sciences ☐ Behavioural & social sciences ☐ Ecological, evolutionary & environmental sciences

For a reference copy of the document with all sections, see nature.com/documents/nr-reporting-summary-flat.pdf

Life sciences study design

All studies must disclose on these points even when the disclosure is negative.

Sample size	In this study, we assessed ribociclib activity in a cohort of 37 patient-derived xenograft models from primary/metastatic breast cancer patients, according to availability. For in vivo experiments, sample size was determined using the Resource equation method (Mead 1988). For ABC-POP trial, a total of 100 patients were to be randomized in a 3:1 allocation to provide a 92% power to detect a difference in the proportion of patients with antiproliferative response from 5% (control) to a 35% (intervention) using a X^2 test with a pooled variance estimation at 0.05 significance level.
Data exclusions	No data was excluded from the analysis in this article.
Replication	In vivo experiments were performed once with the indicated biological replicates. In vitro experiments were performed at least three independent times. Exceptionally, some experimental conditions are shown by duplicate due to lack of biological material availability. All attempts at replication were successful.
Randomization	For in vivo experiments, tumor-bearing mice were randomly allocated into treatments groups with tumors ranging 100-300 mm3 (for drug efficacy experiments) or 500 mm3 (for short-term pharmacodynamic experiments). For in vitro experiments, experimental groups were allocated in sequential order. For the ABC-POP trial, patients (n=105) were randomized 3:1 to abemaciclib 150mg twice daily for 14 days vs. no treatment.
Blinding	The investigators were blinded to group allocation during data collection and analysis.

Behavioural & social sciences study design

All studies must disclose on these points even when the disclosure is negative.

Study description	*Briefly describe the study type including whether data are quantitative, qualitative, or mixed-methods (e.g. qualitative cross-sectional, quantitative experimental, mixed-methods case study).*
Research sample	*State the research sample (e.g. Harvard university undergraduates, villagers in rural India) and provide relevant demographic information (e.g. age, sex) and indicate whether the sample is representative. Provide a rationale for the study sample chosen. For studies involving existing datasets, please describe the dataset and source.*
Sampling strategy	*Describe the sampling procedure (e.g. random, snowball, stratified, convenience). Describe the statistical methods that were used to predetermine sample size OR if no sample-size calculation was performed, describe how sample sizes were chosen and provide a rationale for why these sample sizes are sufficient. For qualitative data, please indicate whether data saturation was considered, and what criteria were used to decide that no further sampling was needed.*
Data collection	*Provide details about the data collection procedure, including the instruments or devices used to record the data (e.g. pen and paper, computer, eye tracker, video or audio equipment) whether anyone was present besides the participant(s) and the researcher, and whether the researcher was blind to experimental condition and/or the study hypothesis during data collection.*
Timing	*Indicate the start and stop dates of data collection. If there is a gap between collection periods, state the dates for each sample cohort.*
Data exclusions	*If no data were excluded from the analyses, state so OR if data were excluded, provide the exact number of exclusions and the rationale behind them, indicating whether exclusion criteria were pre-established.*
Non-participation	*State how many participants dropped out/declined participation and the reason(s) given OR provide response rate OR state that no participants dropped out/declined participation.*
Randomization	*If participants were not allocated into experimental groups, state so OR describe how participants were allocated to groups, and if allocation was not random, describe how covariates were controlled.*

Ecological, evolutionary & environmental sciences study design

All studies must disclose on these points even when the disclosure is negative.

Study description	*Briefly describe the study. For quantitative data include treatment factors and interactions, design structure (e.g. factorial, nested, hierarchical), nature and number of experimental units and replicates.*
Research sample	*Describe the research sample (e.g. a group of tagged Passer domesticus, all Stenocereus thurberi within Organ Pipe Cactus National Monument), and provide a rationale for the sample choice. When relevant, describe the organism taxa, source, sex, age range and any manipulations. State what population the sample is meant to represent when applicable. For studies involving existing datasets, describe the data and its source.*
Sampling strategy	*Note the sampling procedure. Describe the statistical methods that were used to predetermine sample size OR if no sample-size calculation was performed, describe how sample sizes were chosen and provide a rationale for why these sample sizes are sufficient.*
Data collection	*Describe the data collection procedure, including who recorded the data and how.*
Timing and spatial scale	*Indicate the start and stop dates of data collection, noting the frequency and periodicity of sampling and providing a rationale for these choices. If there is a gap between collection periods, state the dates for each sample cohort. Specify the spatial scale from which the data are taken*
Data exclusions	*If no data were excluded from the analyses, state so OR if data were excluded, describe the exclusions and the rationale behind them, indicating whether exclusion criteria were pre-established.*
Reproducibility	*Describe the measures taken to verify the reproducibility of experimental findings. For each experiment, note whether any attempts to repeat the experiment failed OR state that all attempts to repeat the experiment were successful.*
Randomization	*Describe how samples/organisms/participants were allocated into groups. If allocation was not random, describe how covariates were controlled. If this is not relevant to your study, explain why.*
Blinding	*Describe the extent of blinding used during data acquisition and analysis. If blinding was not possible, describe why OR explain why blinding was not relevant to your study.*

Did the study involve field work? ☐ Yes ☐ No

Field work, collection and transport

Field conditions	*Describe the study conditions for field work, providing relevant parameters (e.g. temperature, rainfall).*
Location	*State the location of the sampling or experiment, providing relevant parameters (e.g. latitude and longitude, elevation, water depth).*
Access & import/export	*Describe the efforts you have made to access habitats and to collect and import/export your samples in a responsible manner and in compliance with local, national and international laws, noting any permits that were obtained (give the name of the issuing authority, the date of issue, and any identifying information).*
Disturbance	*Describe any disturbance caused by the study and how it was minimized.*

Reporting for specific materials, systems and methods

We require information from authors about some types of materials, experimental systems and methods used in many studies. Here, indicate whether each material, system or method listed is relevant to your study. If you are not sure if a list item applies to your research, read the appropriate section before selecting a response.

Materials & experimental systems

n/a	Involved in the study	
☐	☒	Antibodies
☐	☒	Eukaryotic cell lines
☒	☐	Palaeontology and archaeology
☐	☒	Animals and other organisms
☐	☒	Human research participants
☒	☐	Clinical data
☒	☐	Dual use research of concern

Methods

n/a	Involved in the study	
☒	☐	ChIP-seq
☒	☐	Flow cytometry
☒	☐	MRI-based neuroimaging

Antibodies

Antibodies used

Primary antibodies used for immunohistochemistry (IHC) were cyclin D1 (RM9104, 1:100) from ThermoScientific; pRb (554136, 1:100) from BD Pharmigen; phospho-pRb S807/811 (8516, 1:300) from Cell Signaling Technology; cyclin E1 (05-363, 1:300) from Millipore; p16 (725-4713), ER (790-4324), PR (790-2223), Ki67 (790-4286) and HER2 (790-2991) from Ventana Medical Systems, Roche. Primary antibodies used for immunofluorescence (IF) were Cytokeratin 18 (ab133263; 1:500) and Alexa Fluor® 568 anti-Vimentin (ab202504; 1:500) both from Abcam and secondary antibody was Alexa Fluor® 488 goat anti-rabbit IgG (A48282, 1:2000). Primary antibodies used for Western blot were CDK4 (12790), cyclin D2 (3741), phospho-pRb S807/811 (9308), phospho-pRb S780 (9307), phospho-CDK2 T160 (2561), phospho-AKT T308 (2965), phospho-AKT S473 (9271), AKT (9272), PARP (9542) and FGFR1 (3472) from Cell Signaling Technology; CDK6 (ab124821), cyclin D1 (ab40754) and cyclin E2 (ab40890) from Abcam; Tubulin (T-9026) from Sigma; cyclin E1 (sc-481), CDK2 (sc-163) and, human GAPDH (sc137179) from Santa Cruz Biotechnology; p16-INKA (10883-I-AP) from ProteinTech; pRb (554136) from BD Pharmigen; ER-alpha (MS-315-P0) from Neomarkers. All primary antibodies were diluted 1:1000 except for human GAPDH and Tubulin that were diluted 1:5000. Secondary antibodies used for Western blot were goat anti-rabbit IgG HRP linked whole antibody (NA934) and goat anti-mouse IhG HRP linked whole antibody (NA931) from Sigma-Aldrich. All secondary antibodies were diluted 1:2000.

Validation

Antibodies for WB
-Anti-CDK4 (D9G3E) Rabbit mAb CST #12790: CDK4 (D9G3E) Rabbit mAb #12790 recognizes endogenous levels of total CDK4 protein. The antibody presents reactivity for human and monkey species. The antibody has been validated for western blotting, immunohistochemistry, immunofluorescence and flow cytometry. Jurkat, Hela, MCF7 and COS-7 cell lines were used to validate western blotting application. Paraffin-embedded human breast and lung carcinoma samples were used to validate immunohistochemistry application. MCF7 cell line was used to validate immunofluorescence application. Jurkat cell line was used to validate flow cytometry application. Link: https://www.cellsignal.com/products/primary-antibodies/cdk4-d9g3e-rabbit-mab/12790
-Anti-Cyclin D2 (D52F9) Rabbit mAb CST #3741: Cyclin D2 (D52F9) Rabbit mAb detects endogenous levels of total cyclin D2 protein. The antibody presents reactivity for human, mouse and rat species. The antibody has been validated for western blotting and immunoprecipitation. U-2 OS, ARCHIN, RD and YB2/0 cell lines were used to validate western blotting application. Link: https://www.cellsignal.com/products/primary-antibodies/cyclin-d2-d52f9-rabbit-mab/3741
-Anti-Phospho-Rb (Ser807/811) Rabbit pAb CST #9308: Phospho-Rb (Ser807/811) Rabbit pAb detects endogenous levels of Rb when phosphorylated at serine 807/811. The antibody may cross-react with Rb phosphorylated at Ser608. The antibody presents reactivity for human, mouse and rat species. Based on sequence homology, it is predicted that the antibody would present reactivity for mouse species. The antibody has been validated for western blotting and immunoprecipitation. Human fibroblast cells were used to validate western blotting application. Link: https://www.cellsignal.com/products/primary-antibodies/phospho-rb-ser807-811-antibody/9308
-Anti-Phospho-Rb (Ser780) Rabbit pAb CST #9307: Phospho-Rb (Ser780) Rabbit pAb detects endogenous levels of Rb only when phosphorylated at Ser780. The antibody presents reactivity for human, rat and monkey species. The antibody has been validated for western blotting and immunoprecipitation. Human fibroblast cells were used to validate western blotting application. Link: https://www.cellsignal.com/products/primary-antibodies/phospho-rb-ser780-antibody/9307
-Anti-Phospho-CDK2 (Thr160) Rabbit pAb CST #2561: Phospho-CDK2 (Thr160) Rabbit pAb detects endogenous levels of CDK2 only when phosphorylated at threonine 160. The antibody weakly cross-reacts with cdc2 phosphorylated at Thr161. The antibody presents reactivity for human, mouse and rat species. The antibody has been validated for western blotting, immunoprecipitation and flow cytometry. HeLa cells were used to validate western blotting application. Jurkat cell line was used to validate flow cytometry application. Link: https://www.cellsignal.com/products/primary-antibodies/phospho-cdk2-thr160-antibody/2561
-Anti-Phospho-Akt (Thr308) (C31E5E) Rabbit mAb CST #2965: Phospho-Akt (Thr308) (C31E5E) Rabbit mAb detects endogenous levels of Akt only when phosphorylated at Thr308. The antibody presents reactivity for human, mouse, rat, hamster and monkey species. The antibody has been validated for western blotting. NIH/3T3 and Jurkat cells were used to validate western blotting application. Link: https://www.cellsignal.com/products/primary-antibodies/phospho-akt-thr308-c31e5e-rabbit-mab/2965
-Anti-Phospho-Akt (Ser473) Rabbit pAb CST #9271: Phospho-Akt (Ser473) Rabbit pAb detects endogenous levels of Akt1 only when phosphorylated at Ser473. This antibody also recognizes Akt2 and Akt3 when phosphorylated at the corresponding residues. The antibody presents reactivity for human, mouse, rat, hamster, monkey, D. melanogaster, bovine and dog species. Based on sequence homology, it is predicted that the antibody would present reactivity for monkey, chicken, xenopus and horse species. The antibody has been validated for western blotting, immunoprecipitation, immunofluorescence and flow cytometry. NIH/3T3 cells were used to validate western blotting application. HEK293 cells were used to validate immunoprecipitation application. C2C12 cells were used to validate immunofluorescence application. LNCaP cells were used to validate flow cytometry application. Link: https://www.cellsignal.com/products/primary-antibodies/phospho-akt-ser473-antibody/9271
-Anti-Akt Rabbit pAb CST #9272: Akt Rabbit pAb detects endogenous levels of total Akt1, Akt2 and Akt3 proteins. The antibody presents reactivity for human, mouse, rat, hamster, monkey, chicken, D. melanogaster, bovine, dog, pig and guinea pig species. The antibody has been validated for western blotting, immunoprecipitation, immunofluorescence and flow cytometry. CHO, HeLa and NIH/3T3 cells were used to validate western blotting application. C2C12 cells were used to validate immunofluorescence application. Jurkat cells were used to validate flow cytometry application. Link: https://www.cellsignal.com/products/primary-antibodies/akt-antibody/9272?site-search-type=Products&N=4294956287&Ntt=akt+%289272%29%2C+&fromPage=plp&_requestid=518831
-Anti-PARP Rabbit pAb CST #9542: PARP Rabbit pAb detects endogenous levels of full length PARP1 (116 kDa), as well as the large fragment (89 kDa) of PARP1 resulting from caspase cleavage. The antibody presents reactivity for human, mouse, rat and monkey species. The antibody has been validated for western blotting. NIH/3T3 cells were used to validate western blotting application. Link: https://www.cellsignal.com/products/primary-antibodies/parp-antibody/9542
-Anti-FGF Receptor 1 Rabbit pAb CST #3472: FGF Receptor 1 Rabbit pAb detects transfected levels of FGF receptor 1. The antibody presents reactivity for human. Based on sequence homology, it is predicted that the antibody would present reactivity for mouse and rat species. The antibody has been validated for western blotting and immunoprecipitation. COS cells were used to validate western blotting application. Link: https://www.cellsignal.com/products/primary-antibodies/fgf-receptor-1-antibody/3472
-Anti-Cdk6 Rabbit mAb [EPR4515] (ab124821): Anti-Cdk6 Rabbit mAb [EPR4515] detects endogenous levels of total CDK6 protein. The antibody presents reactivity for human species. The antibody has been validated for western blotting, immunohistochemistry, immunofluorescence and flow cytometry. Jurkat, K562, HAP1, HeLa and 293T cell lines were used to validate western blotting application. Human tonsil tissue was used to validate immunohistochemistry application. HeLa and wild-type HAP1 cells were used to validate flow cytometry applications. Link: https://www.abcam.com/cdk6-antibody-epr4515-ab124821.html?productWallTab=ShowAll
-Anti-Cyclin D1 Rabbit mAb [EP272Y] (ab40754): Anti-Cyclin D1 Rabbit mAb [EP272Y] detects endogenous levels of total Cyclin D1

protein. The antibody presents reactivity for human and mouse species. Based on homology, it is predicted that the antibody would present reactivity for rat species. The antibody has been validated for western blotting, immunohistochemistry and immunofluorescence. HAP1, A431, Hap1, HeLa and MCF7 cell lines were used to validate western blotting application. Human kidney carcinoma and human hepatocellular carcinoma tissues were used to validate immunohistochemistry application. MCF7 cell line was used to validate immunofluorescence application. Link: https://www.abcam.com/cyclin-d1-antibody-ep272y-ab40754.html
-Anti-Cyclin E2 Rabbit mAb [EP454Y] (ab40890): Recombinant Anti-Cyclin E2 Rabbit mAb [EP454Y] detects endogenous levels of total Cyclin E2 protein. The antibody presents reactivity for human species. The antibody has been validated for western blotting, immunoprecipitation, immunohistochemistry, immunofluorescence and flow cytometry. Wild-type HAP1, MCF7, HeLa, and Jurkat cell lines were used to validate western blotting application. Jurkat cell line was used to validate immunoprecipitation application. Human breast carcinoma tissue was used to validate immunohistochemistry application. HeLa cell line was used to validate immunofluorescence application. HeLa cell line was used to validate flow cytometry application. Link: https://www.abcam.com/cyclin-e2-antibody-ep454y-ab40890.html
-Anti-α-Tubulin Mouse mAb (SIGMA T9026): Anti-α-Tubulin Mouse mAb (T9026) detects total levels of α-tubulin protein. The antibody presents reactivity for bovine, rat, yeast, human, mouse, chicken, fungi, amphibian species. The antibody has been validated for western blotting, immunohistochemistry and immunofluorescence. Human osteosarcoma and in breast cancer cell lines were used to validate western blotting application. HeLa cell line was used to validate immunofluorescence application. Xenopus embryos were used to validate immunohistochemistry application. Link: https://www.sigmaaldrich.com/ES/es/product/sigma/t9026
-Anti-Cyclin E Rabbit mAb (M-20) (sc-481): Anti-Cyclin E Rabbit mAb (M-20) detects total levels of cyclin E1 protein. The antibody presents reactivity for mouse, rat and human species. The antibody has been validated for western blotting, immunoprecipitation, immunofluorescence, immunohistochemistry and solid phase ELISA. 3611-RF, Jurkat, KNRK cell lines were used to validate western blotting, immunoprecipitation, immunofluorescence, immunohistochemistry and solid phase ELISA applications. Link: https://datasheets.scbt.com/sc-481.pdf / https://www.scbt.com/p/cyclin-e-antibody-m-20
-Anti-CDK2 Rabbit pAb (M2) (sc-163): Anti-CDK2 Rabbit pAb (M2) (sc-163) detects total levels of CDK2 protein. The antibody presents reactivity for mouse, rat, human, hamster, equine, canine, bovine and porcine species. The antibody has been validated for western blotting, immunoprecipitation, immunofluorescence and solid phase ELISA. 293T and NAMALWA cell lines were used to validate western blotting, immunoprecipitation, immunofluorescence and solid phase ELISA applications. Link: https://datasheets.scbt.com/sc-163.pdf / https://www.scbt.com/p/cdk2-antibody-m2
-Anti-GAPDH Mouse mAb (A-3) (sc-137179): Anti-GAPDH Mouse mAb (A-3) (sc-137179) detects total levels of GAPDH and GAPDH-2 protein. The antibody presents reactivity for mouse, rat and human species. The antibody has been validated for western blotting, immunoprecipitation, immunofluorescence, immunohistochemistry and solid phase ELISA. HeLa, Jurkat and NIH/3T3 cell lines were used to validate western blotting, immunoprecipitation, immunofluorescence, immunohistochemistry and solid phase ELISA applications. Link: https://datasheets.scbt.com/sc-137179.pdf / https://www.scbt.com/p/gapdh-antibody-a-3?productCanUrl=gapdh-antibody-a-3&_requestid=490002
-Anti-p16-INK4A Rabbit pAb (Proteintech 10883-I-AP): Anti-p16-INK4A Rabbit pAb detects total levels of p16-INK4A protein. The antibody presents reactivity for human and monkey species. The antibody has been validated for western blotting, immunoprecipitation, immunofluorescence and immunohistochemistry. HEK293, HeLa, HepG2, PC-3 cell lines were used to validate western blotting. HEK293 cell line was used to validate immunoprecipitation application. Human cervical cancer tissue was used to validate immunohistochemistry application. HeLa cell line was used to validate immunofluorescence application. Link: https://www.ptglab.com/products/P16,P19-Antibody-10883-1-AP.htm
-Anti-Human Retinoblastoma protein Mouse mAb (G3-245) (BD Pharmingen 554136): Anti-Human Retinoblastoma protein Mouse mAb (G3-245) detects total levels of retinoblastoma protein. The antibody presents reactivity for human, monkey, mouse, rat, mink and quail species. The antibody has been validated for western blotting, immunoprecipitation, immunohistochemistry and flow cytometry. MOLT-4, A549, U-2 OS and HeLa cell lines were used to validate western blotting, immunoprecipitation, immunohistochemistry and flow cytometry applications. Link: https://www.bdbiosciences.com/en-es/products/reagents/flow-cytometry-reagents/research-reagents/single-color-antibodies-ruo/purified-mouse-anti-human-retinoblastoma-protein.554136
-Anti-Estrogen receptor Mouse mAb Ab-10 (Clone TE111.5D11) (ThermoFisher #MS-315-P0): Anti-Estrogen receptor Mouse mAb Ab-10 detects total levels of estrogen receptor protein. The antibody presents reactivity for human, cow, pig, dog, mouse, rat, sheep, rabbit, hamster, and chicken species. The antibody has been validated for western blotting and immunoprecipitation. T47D cell line was used to validate western blotting and immunoprecipitation applications. Link: https://assets.thermofisher.com/TFS-Assets/APD/Specification-Sheets/D11757.pdf
Antibodies for immunohistochemistry
-Anti-Cyclin D1 Rabbit mAb (Clone SP4) (ThermoFisher #RM-9104-S0): Anti-Cyclin D1 Rabbit mAb detects total levels of cyclin D1 protein. The antibody presents reactivity for human species. The antibody has been validated for western blotting and immunohistology. Breast carcinoma and mantle cell lymphoma were used to validate immunohistology application. Link: https://tools.thermofisher.com/content/sfs/brochures/D12533~.pdf
-Anti-Human Retinoblastoma protein Mouse mAb (G3-245) (BD Pharmingen 554136): Anti-Human Retinoblastoma protein Mouse mAb (G3-245) detects total levels of retinoblastoma protein. The antibody presents reactivity for human, monkey, mouse, rat, mink and quail species. The antibody has been validated for western blotting, immunoprecipitation, immunohistochemistry and flow cytometry. MOLT-4, A549, U-2 OS and HeLa cell lines were used to validate western blotting, immunoprecipitation, immunohistochemistry and flow cytometry applications. Link: https://www.bdbiosciences.com/en-es/products/reagents/flow-cytometry-reagents/research-reagents/single-color-antibodies-ruo/purified-mouse-anti-human-retinoblastoma-protein.554136
-Anti-Phospho-Rb (Ser807/811) (D20B12) XP® Rabbit mAb CST #8516: Phospho-Rb (Ser807/811) (D20B12) XP® Rabbit mAb recognizes endogenous levels of Rb protein only when phosphorylated at Ser807, Ser811, or at both sites. This antibody does not cross-react with Rb phosphorylated at Ser608. The antibody presents reactivity for human, mouse, rat and monkey species. The antibody has been validated for western blotting, immunoprecipitation, immunohistochemistry, immunofluorescence and flow cytometry. MCF7 and WI-38 cell lines were used to validate western blotting application. Cos cell line was used to validate immunoprecipitation application. Human colon, lung and ovarian carcinoma and mouse spleen tissues were used to validate immunohistochemistry application. MCF7 and BT-549 cell lines were used to validate immunofluorescence application. Jurkat cell line was used to validate flow cytometry application. Link: https://www.cellsignal.com/products/primary-antibodies/phospho-rb-ser807-811-d20b12-xp-rabbit-mab/8516
-Anti-Cyclin E Mouse mAb (Clone HE12) (Millipore 05-363): Anti-Cyclin E Mouse mAb (Clone HE12) detects total levels of cyclin E protein. The antibody presents reactivity for human species. The antibody has been validated for western blotting, immunoprecipitation and immunohistochemistry. Human A431 cell line was used to validate western blotting application. Link: https://www.merckmillipore.com/ES/es/product/Anti-Cyclin-E-Antibody-clone-HE12,MM_NF-05-363?ReferrerURL=https%3A%2F%2Fwww.google.com%2F#anchor_Applications
-Anti-p16INK4a (E6H4) Mouse mAb (Ventana 805-4713): Anti-p16INK4a (E6H4) Mouse mAb detects total levels of p16INK4a protein.

The antibody presents reactivity for human species. The antibody has been validated for immunohistochemistry. Normal human tissues were used to validate immunohistochemistry application. Link: https://pim-eservices.roche.com/eLD/api/downloads/f1ce6d58-2236-ea11-fc90-005056a71a5d?countryIsoCode=pi
-Anti-Estrogen receptor (ER) Rabbit mAb (SP1) (Ventana 790-4324): Anti-Estrogen receptor Rabbit mAb (SP1) detects total levels of human estrogen alpha receptor protein. The antibody presents reactivity for human species. The antibody has been validated for immunohistochemistry. Multiple normal human tissues were used to validate immunohistochemistry application. Link: https://pim-eservices.roche.com/eLD/api/downloads/2b989f55-4533-ea11-fc90-005056a71a5d?countryIsoCode=pi
-Anti-Progesterone Receptor (PR) Rabbit mAb (1E2) (Ventana 790-2223): Anti-Progesterone Receptor (PR) Rabbit mAb (1E2) detects A, and B isoforms of human progesterone receptor (PR) protein. The antibody presents reactivity for human species. The antibody has been validated for immunohistochemistry. Multiple normal human tissues were used to validate immunohistochemistry application. Link: https://pim-eservices.roche.com/eLD/api/downloads/76ea4fea-e112-ea11-fa90-005056a772fd?countryIsoCode=pi
-Anti-Ki-67 Rabbit mAb (30-9) (Ventana 790-4286): Anti-Ki-67 Rabbit mAb (30-9) detects proliferation Ki-67 (MKI67), more commonly known as Ki-67. The antibody presents reactivity for human species. The antibody has been validated for immunohistochemistry. Multiple normal human tissues were used to validate immunohistochemistry application. Link: https://pim-eservices.roche.com/eLD/api/downloads/fa95325e-7133-ea11-fa90-005056a772fd?countryIsoCode=pi
-Anti-HER-2/neu Rabbit mAb (4B5) (Ventana 790-2991): Anti-HER-2/neu Rabbit mAb (4B5) detects total levels of the transmembrane human epidermal growth factor receptor 2 (HER2) protein. The antibody presents reactivity for human species. The antibody has been validated for immunohistochemistry. Multiple normal human tissues were used to validate immunohistochemistry application. Link: https://pim-eservices.roche.com/eLD/api/downloads/8b127bac-6533-ea11-fa90-005056a772fd?countryIsoCode=pi

Antibodies for immunofluorescence
-Anti-Cytokeratin 18 Rabbit mAb [EPR1626] (ab133263): Anti-Cytokeratin 18 Rabbit mAb [EPR1626] detects endogenous levels of total cytokeratin 18. The antibody presents reactivity for human and rat species. The antibody has been validated for western blotting, flow cytometry, immunohistochemistry and immunofluorescence. HeLa, MCF7, Jurkat and A431 cell lines were used to validate western blotting application. Human endometrium, human gastric adenocarcinoma tissue, human breast tissue, and rat liver tissues were used to validate immunohistochemistry application. HeLa cell line was used to validate immunofluorescence and flow cytometry applications. Link: https://www.abcam.com/cytokeratin-18-antibody-epr1626-ab133263.html
-Alexa Fluor® 568 Anti-Vimentin Rabbit mAb [EPR3776] (ab202504): Alexa Fluor® 568 Anti-Vimentin Rabbit mAb [EPR3776] detects endogenous levels of total vimentin. The antibody presents reactivity for human and mouse species. Based on homology, it is predicted that the antibody would present reactivity for rat species. The antibody has been validated for immunofluorescence and flow cytometry. NIH3T3, HeLa and wildtype HAP1 cell lines were used to validate immunofluorescence application. Wildtype HAP1 cell line was used to validate flow cytometry application. Link: https://www.abcam.com/alexa-fluor-568-vimentin-antibody-epr3776-cytoskeleton-marker-ab202504.html

Eukaryotic cell lines

Policy information about cell lines

Cell line source(s)	293T (CRL-3216), MCF7 (HTB-22) and T47D (HTB-133) cell lines were obtained from ATCC
Authentication	Cell lines were authenticated utilizing short tandem repeat (STR) profiling (FTA Sample Collection Kit for Human Cell Authentication Service; ATCC services)
Mycoplasma contamination	Mycoplasma test was performed every 5 passages (MycoAlert™ Mycoplasma Detection Kit; LONZA)
Commonly misidentified lines (See ICLAC register)	No misidentified cells lines were employed to obtain the findings described in this article

Palaeontology and Archaeology

Specimen provenance	*Provide provenance information for specimens and describe permits that were obtained for the work (including the name of the issuing authority, the date of issue, and any identifying information). Permits should encompass collection and, where applicable, export.*
Specimen deposition	*Indicate where the specimens have been deposited to permit free access by other researchers.*
Dating methods	*If new dates are provided, describe how they were obtained (e.g. collection, storage, sample pretreatment and measurement), where they were obtained (i.e. lab name), the calibration program and the protocol for quality assurance OR state that no new dates are provided.*

☐ Tick this box to confirm that the raw and calibrated dates are available in the paper or in Supplementary Information.

Ethics oversight	*Identify the organization(s) that approved or provided guidance on the study protocol, OR state that no ethical approval or guidance was required and explain why not.*

Note that full information on the approval of the study protocol must also be provided in the manuscript.

Animals and other organisms

Policy information about studies involving animals; ARRIVE guidelines recommended for reporting animal research

Laboratory animals	The laboratory animales employed in this article were six-week-old female athymic nude HsdCpb:NMRI-Foxn1nu mice (Janvier) and

Laboratory animals	NOD.Cg-PrkdcscidIl2rgtm1Wjl/SzJ mice (Charles Rives). Mice were housed in air-filtered flow cabinets with a 12-hours light cycle at 18-23ºC, 40-60% of humidity and food and water ad libitum
Wild animals	No wild animals were employed in this scientific article.
Field-collected samples	No field-collected samples were employed in this scientific article.
Ethics oversight	All animal procedures were approved by the Ethics Committee of Animal Research of the Vall d´Hebron Institute of Oncology, the Catalan Government (FUE-2020-01541918), the National Research Ethics Service, Cambridgeshire 2 REC (RED reference number: 08/H0308/178 and http://caldaslab.crik.cam.ac.uk/bcape/) and by the Central Office for Research Ethics Committee study number 05/Q1402/25. All of them were conformed to the European Union's animal care directive (2010/63/EU).

Note that full information on the approval of the study protocol must also be provided in the manuscript.

Human research participants

Policy information about studies involving human research participants

Population characteristics	Breast cancer patients with early-breast cancer or metastatic breast cancer disease
Recruitment	Untreated females aged 18 years or older who signed written informed consent and were diagnosed with HR-positive, non-metastatic invasive breast carcinoma (ABC-POP trial).<br>HR-positive breast cancer metastatic patients aged 18 years or older treated at the Vall d'Hebron University Hospital with CDK4/6 inhibitors in monotherapy or combination with available tumor tissue sample who signed an IRB-approved informed consent.
Ethics oversight	The clinical trial were conducted according to the principles of Good Clinical Practice and the Declaration of Helsinki; ABC-POP trial was approved by an institutional review board (CSET 2013: 2016) and by a National Ethics Committee (Eudract 2013-002967-24, 25/09/2013). The Ethics Committee for Clinical Research of Vall d'Hebron Hospital approved sample collection from breast cancer patients of Vall d'Hebron Hospital (PR(AG)130/2015).

Note that full information on the approval of the study protocol must also be provided in the manuscript.

Clinical data

Policy information about clinical studies

All manuscripts should comply with the ICMJE guidelines for publication of clinical research and a completed CONSORT checklist must be included with all submissions.

Clinical trial registration	*Provide the trial registration number from ClinicalTrials.gov or an equivalent agency.*
Study protocol	*Note where the full trial protocol can be accessed OR if not available, explain why.*
Data collection	*Describe the settings and locales of data collection, noting the time periods of recruitment and data collection.*
Outcomes	*Describe how you pre-defined primary and secondary outcome measures and how you assessed these measures.*

Dual use research of concern

Policy information about dual use research of concern

Hazards

Could the accidental, deliberate or reckless misuse of agents or technologies generated in the work, or the application of information presented in the manuscript, pose a threat to:

No	Yes	
☒	☐	Public health
☒	☐	National security
☒	☐	Crops and/or livestock
☒	☐	Ecosystems
☒	☐	Any other significant area

Experiments of concern

Does the work involve any of these experiments of concern:

No	Yes	
☒	☐	Demonstrate how to render a vaccine ineffective
☒	☐	Confer resistance to therapeutically useful antibiotics or antiviral agents
☒	☐	Enhance the virulence of a pathogen or render a nonpathogen virulent
☒	☐	Increase transmissibility of a pathogen
☒	☐	Alter the host range of a pathogen
☒	☐	Enable evasion of diagnostic/detection modalities
☒	☐	Enable the weaponization of a biological agent or toxin
☒	☐	Any other potentially harmful combination of experiments and agents

ChIP-seq

Data deposition

☐ Confirm that both raw and final processed data have been deposited in a public database such as GEO.

☐ Confirm that you have deposited or provided access to graph files (e.g. BED files) for the called peaks.

Data access links<br>*May remain private before publication.*	*For "Initial submission" or "Revised version" documents, provide reviewer access links. For your "Final submission" document, provide a link to the deposited data.*
Files in database submission	*Provide a list of all files available in the database submission.*
Genome browser session<br>(e.g. UCSC)	*Provide a link to an anonymized genome browser session for "Initial submission" and "Revised version" documents only, to enable peer review. Write "no longer applicable" for "Final submission" documents.*

Methodology

Replicates	*Describe the experimental replicates, specifying number, type and replicate agreement.*
Sequencing depth	*Describe the sequencing depth for each experiment, providing the total number of reads, uniquely mapped reads, length of reads and whether they were paired- or single-end.*
Antibodies	*Describe the antibodies used for the ChIP-seq experiments; as applicable, provide supplier name, catalog number, clone name, and lot number.*
Peak calling parameters	*Specify the command line program and parameters used for read mapping and peak calling, including the ChIP, control and index files used.*
Data quality	*Describe the methods used to ensure data quality in full detail, including how many peaks are at FDR 5% and above 5-fold enrichment.*
Software	*Describe the software used to collect and analyze the ChIP-seq data. For custom code that has been deposited into a community repository, provide accession details.*

Flow Cytometry

Plots

Confirm that:

☐ The axis labels state the marker and fluorochrome used (e.g. CD4-FITC).

☐ The axis scales are clearly visible. Include numbers along axes only for bottom left plot of group (a 'group' is an analysis of identical markers).

☐ All plots are contour plots with outliers or pseudocolor plots.

☐ A numerical value for number of cells or percentage (with statistics) is provided.

Methodology

Sample preparation	*Describe the sample preparation, detailing the biological source of the cells and any tissue processing steps used.*
Instrument	*Identify the instrument used for data collection, specifying make and model number.*

Software	*Describe the software used to collect and analyze the flow cytometry data. For custom code that has been deposited into a community repository, provide accession details.*
Cell population abundance	*Describe the abundance of the relevant cell populations within post-sort fractions, providing details on the purity of the samples and how it was determined.*
Gating strategy	*Describe the gating strategy used for all relevant experiments, specifying the preliminary FSC/SSC gates of the starting cell population, indicating where boundaries between "positive" and "negative" staining cell populations are defined.*

☐ Tick this box to confirm that a figure exemplifying the gating strategy is provided in the Supplementary Information.

Magnetic resonance imaging

Experimental design

Design type	*Indicate task or resting state; event-related or block design.*
Design specifications	*Specify the number of blocks, trials or experimental units per session and/or subject, and specify the length of each trial or block (if trials are blocked) and interval between trials.*
Behavioral performance measures	*State number and/or type of variables recorded (e.g. correct button press, response time) and what statistics were used to establish that the subjects were performing the task as expected (e.g. mean, range, and/or standard deviation across subjects).*

Acquisition

Imaging type(s)	*Specify: functional, structural, diffusion, perfusion.*
Field strength	*Specify in Tesla*
Sequence & imaging parameters	*Specify the pulse sequence type (gradient echo, spin echo, etc.), imaging type (EPI, spiral, etc.), field of view, matrix size, slice thickness, orientation and TE/TR/flip angle.*
Area of acquisition	*State whether a whole brain scan was used OR define the area of acquisition, describing how the region was determined.*

Diffusion MRI ☐ Used ☐ Not used

Preprocessing

Preprocessing software	*Provide detail on software version and revision number and on specific parameters (model/functions, brain extraction, segmentation, smoothing kernel size, etc.).*
Normalization	*If data were normalized/standardized, describe the approach(es): specify linear or non-linear and define image types used for transformation OR indicate that data were not normalized and explain rationale for lack of normalization.*
Normalization template	*Describe the template used for normalization/transformation, specifying subject space or group standardized space (e.g. original Talairach, MNI305, ICBM152) OR indicate that the data were not normalized.*
Noise and artifact removal	*Describe your procedure(s) for artifact and structured noise removal, specifying motion parameters, tissue signals and physiological signals (heart rate, respiration).*
Volume censoring	*Define your software and/or method and criteria for volume censoring, and state the extent of such censoring.*

Statistical modeling & inference

Model type and settings	*Specify type (mass univariate, multivariate, RSA, predictive, etc.) and describe essential details of the model at the first and second levels (e.g. fixed, random or mixed effects; drift or auto-correlation).*
Effect(s) tested	*Define precise effect in terms of the task or stimulus conditions instead of psychological concepts and indicate whether ANOVA or factorial designs were used.*

Specify type of analysis: ☐ Whole brain ☐ ROI-based ☐ Both

Statistic type for inference (See Eklund et al. 2016)	*Specify voxel-wise or cluster-wise and report all relevant parameters for cluster-wise methods.*
Correction	*Describe the type of correction and how it is obtained for multiple comparisons (e.g. FWE, FDR, permutation or Monte Carlo).*

Models & analysis

n/a	Involved in the study
☐	☐ Functional and/or effective connectivity
☐	☐ Graph analysis
☐	☐ Multivariate modeling or predictive analysis

Functional and/or effective connectivity

Report the measures of dependence used and the model details (e.g. Pearson correlation, partial correlation, mutual information).

Graph analysis

Report the dependent variable and connectivity measure, specifying weighted graph or binarized graph, subject- or group-level, and the global and/or node summaries used (e.g. clustering coefficient, efficiency, etc.).

Multivariate modeling and predictive analysis

Specify independent variables, features extraction and dimension reduction, model, training and evaluation metrics.
